# Medical Books and General Readers

**Published:** 1886-11-13

**Authors:** 


### MEDICAL BOOKS AND GENERAL READERS.

*An Alteration Needed.*

How narrow is the range of medical literature, and how short-lived the fame of the authors of it! The very name of Hippocrates is unknown to the unlearned, and the vast majority of the learned are content to know no more than his name. And yet medicine is as old as man, and is in a sense the science of man. It is a curious reflection for the scientific materialist that whilst the profoundest moral and metaphysical ideas have sooner or later always filtered down to, and become the possessions of the masses, the specutions of the physiologist, the pathologist, and the learned practitioner of the medical art have had no interest except for the esoteric few. That is not as it should be, and, it is to be hoped, not as it will be. Metaphysicians and religious teachers have claimed for the thinking part of man the highest pre-eminence. Though this position is not here challenged, a plea is entered for some consideration for the more purely material part. Both for its own sake, and as the casket of the thinking principle, the human body is worthy of the most diligent and reverent regard. Medical literature concerns itself with the human body; with that body both in its normal and abnormal states; both in health and disease. It cannot but be important—it ought to be interesting—to all men to know something of their bodies, those parts of themselves which are the most obvious, and which have so very much to do with their happiness or their misery, their failure or their success in life.

*Why Medical Books are Unpopular.*

It is of course easy to understand why medical books never have been and never can be popular. They are technical to the last degree, not merely in their terminology, but also in their principles and purpose. Nevertheless, a great service would be rendered to the general reader if he were enabled to understand the fundamental ideas on which modern medicine is based, and could be made acquainted from time to time with its scientific and practical progress. It will be part of the business of The Hospital to lend a helping-hand in this direction. Books already written are available for the purpose, and new books as they are published will be carefully and thoroughly reviewed in a popular style. By this means, everyone who is scientifically curious, or who desires medical knowledge for practical purposes, will be furnished with a guiding light whereby he may steer his way intelligently and safely through the maze and darkness.

*A Word of Caution.*

A word of caution may fitly be addressed to those who desire to enter upon this line of inquiry. It must be remembered that there are medical books and medical books. Medical literature may indeed be separated into three divisions. There are books, properly so called, consisting of the solid accumulations of years of original labour and thought in the fields of science and practice. These are the gold of medical literature. Then there are the gleanings and gatherings of laborious mediocrity, of the men who compile books from the writings of others, not because they are heaven-born authors, but because the public are more likely to consult a man who has "written a book" than one who has not done so. These books, so far as they are current coin at all, belong neither to the gold, nor to the silver, but to the bronze standard. Lastly, there are the books written purely and simply for advertising purposes. These, unhappily, are not always the work of unqualified or "quack" doctors. They are often written in a more or less popular style by registered practitioners, and have no other purpose but that of setting forth the merits and abilities of their designing authors. All such books should be promptly consigned to the waste-paper basket, and their authors as promptly relegated to the limbo of forgotten cheats and knaves.

*A Serious Responsibility.*

It is no slight responsibility which rests upon the public press to help readers in the selection and use of books. In this, as in other functions, candour and justice should be the guiding principles: and the object constantly in view the enlightenment, direction, and advantage of the reader. If these principles be well remembered it should be possible to make the best medical literature as interesting to the thoughtful reader as it is undoubtedly instructive and important. Our aim will be to sift the wheat from the chaff. With this object we shall direct attention to those portions of medical books which it will be advantageous to the general reader to peruse and reflect upon. At the present time no such attempt has been made, possibly because of the many difficulties such a course must entail upon the reviewer. It is, however, the plain duty of a responsible journalist to ensure that the readers of his paper shall have their attention called to every matter of general interest which it concerns them to know. We hope our efforts in this direction may prove helpful to many readers and authors, and so do a practical service to medical literature and the general reader.

